# Influence of Resistance-Inducing Chemical Elicitors against Pine Wilt Disease on the Rhizosphere Microbiome

**DOI:** 10.3390/microorganisms8060884

**Published:** 2020-06-11

**Authors:** Mohamed Mannaa, Gil Han, Hee Won Jeon, Junheon Kim, Namgyu Kim, Ae Ran Park, Jin-Cheol Kim, Young-Su Seo

**Affiliations:** 1 Department of Integrated Biological Science, Pusan National University, Busan 46241, Korea; manna_mohamed@yahoo.com (M.M.); croone@pusan.ac.kr (G.H.); titanic622@pusan.ac.kr (N.K.); 2 Division of Applied Bioscience and Biotechnology, Chonnam National University, Gwangju 61186, Korea; jeon-hw@naver.com (H.W.J.); arpark9@naver.com (A.R.P.); 3 Forest Insect Pests and Diseases Division, National Institute of Forest Science, Seoul 02455, Korea; junheonkim@korea.kr

**Keywords:** methyl salicylic acid, acibenzolar-s-methyl, metagenomic analysis, pine wilt disease, pine wood nematode

## Abstract

Pine wilt disease (PWD) caused by *Bursaphelenchus xylophilus* is a major threat to pine forests worldwide. Induction of resistance is a promising and safe management option that should be investigated in relation to its possible influence on the pine tree ecosystem, including the surrounding microbial communities. In this study, two main resistance-inducing chemical elicitors, methyl salicylic acid (MeSA) and acibenzolar-s-methyl (ASM), were tested for their control efficiency against PWD and their influence on the rhizosphere microbial composition. Foliar treatment of pine seedlings with the chemical elicitors resulted in a reduction in PWD severity, with ASM showing better control efficacy, reaching up to 73% compared to the untreated control. Moreover, bacterial community analysis of the rhizosphere revealed significant changes in several microbial taxa that were present at low relative abundance. In particular, ASM treatment resulted in a significant increase in specific microbial taxa, including members of the *Rhodanobacter, Devosia, Bradyrhizobium, Acidibacter, Mesorhizobium*, and *Hyphomicrobium* genera, which are known to play ecological and plant growth-promoting roles. Furthermore, chitinolytic bacteria were shown to be reduced in response to treatment with both MeSA and ASM. Altogether, the present findings demonstrate the occurrence of significant alterations in several ecologically important microbial taxa after treatment with resistance-inducing chemicals. As compared to MeSA treatment, ASM treatment was more effective at suppressing PWD and resulted in more beneficial changes in rhizosphere microbial composition.

## 1. Introduction

Pine trees are significant sources of forest products, play important environmental roles, and strongly contribute to the beauty of the landscape. Pine wilt disease (PWD) is the most serious threat to pine forests worldwide, causing profound economic and environmental damage [1]. It is caused by the native North American pinewood nematode (PWN), *Bursaphelenchus xylophilus* (Nematoda: Aphelenchoididae), which causes little damage in the native habitat in North America. However, upon introduction to non-native habitats, PWN leads to tremendous damage and high mortality rates [2]. The disease was first reported in Japan in 1971, although the typical symptoms have been observed as early as 1905 [3]. Then, the disease spread to China, South Korea, and Taiwan and, more recently, to Portugal and Spain, still representing a serious threat to all European pine forests [4,5,6].

Although PWN, along with its associated bacteria, was confirmed to be the causative agent, the disease has a complex and, as yet not completely understood, etiology [7]. Following access to the plants through the feeding and oviposition of the pine sawyer longhorn-beetles, Monochamus spp. insect vector, PWN causes the destruction of the plant vascular system [8]. Inside the susceptible host, the nematode can reproduce quickly and lead to xylem dysfunction resulting in discoloration, wilting, and consequent death of host trees [9,10].

The histological and physiological changes occur before the increase in the nematode population, which suggests the pathogenetic role of other etiological agents [7,11]. Moreover, axenic (microbe-free) nematodes were reported to lose their pathogenicity [12]. Apparently, bacteria associated with PWN may have a role in the pathogenicity of the disease by the production of phytotoxins [12,13]. Phenylacetic acid produced by *Bacillus* spp. and pyochelin produced by *Burkholderia arboris*, which are carried by the PWN, were found to possess phytotoxic activity against pine callus and seedlings [14,15]. The roles of the PWN- and pine-associated bacteria are not only limited to the pathogenesis of PWD as they also strongly affect plant growth and defense-related activities. Studies on the diversity and composition of pine endophytic bacteria suggest their important role in the regulation of plant response to PWN, as well as in protective nematotoxic activity [16,17].

Strict quarantine measures and extensive breeding programs for the selection of resistant trees represent the main effective strategies available for controlling the spread of the disease [18,19]. Yet, several methods have been studied and proposed for the management of PWD, including insect vector eradication, physical chipping, and tree injection with nematicidal compounds [20,21,22]. Due to environmental and health risk-related concerns, restrictions have been imposed on the use of nematicides, and the search for safer alternatives such as biological control and induction of resistance, has consequently been encouraged [18,23]. The induction of plant resistance against pathogenic organisms by treatments with biotic or abiotic elicitors is a well-established approach. In pine trees, avirulent nematode isolates were shown to induce physiological changes leading to PWN suppression and decreased disease severity [10]. Recently, Kim et al. [22], isolated pine endophytic bacterial strains and selected three species (*Pseudomonas putida* 16YSM-E48, *Curtobacterium pusillum* 16YSM-P180, and *Stenotrophomonas rhizophila* 16YSM-P39) with the ability to induce systemic resistance against PWD in callus and pine seedlings as was confirmed molecularly and phenotypically.

In general, systemic acquired resistance (SAR) is the most understood and best characterized signal pathway related to broad-spectrum resistance in plants and is mediated by salicylic acid [24]. Salicylic acid or other chemical compounds mimicking its role such as acibenzolar-s-methyl (ASM) and methyl salicylic acid (MeSA), were shown to elicit SAR against many pathogens including fungi, bacteria, viruses, and nematodes, by acting at different points in the defense network [23,24]. ASM was the first resistance elicitor with activity against a broad spectrum of diseases to be commercialized as BION^®^ in Europe and ACTIGARD^®^ in the USA [25]. Although the control efficacy of resistance elicitors is relatively low compared to chemical pesticides, they remain a very important, environmentally safe, alternative, particularly because they lack direct antimicrobial activity, which could induce resistance in specific pathogenic populations [26].

Given the fact that the endophytic and other pine-associated microbial communities play important roles in the progression or suppression of the PWD, the effect of the proposed control methods should be studied within the frame of other ecological variables involved. However, most studies on resistance-inducing elicitors focus on their mechanism of action and control efficacy. Therefore, in this study, two main resistance-inducing chemical elicitors (ASM and MeSA) were tested for their suppressive activity against PWD and their effect on the rhizosphere and root microbiota was studied by metagenomic analysis.

## 2. Materials and Methods

### 2.1. Preparation and Treatment with the Chemical Inducers and Nematode Inoculum

To evaluate the effect of MeSA and ASM on PWD severity and rhizosphere microbial composition, a seedling assay was performed as follows. MeSA (>99.0% purity) was purchased from Tokyo Chemical Industry Co., Ltd. (Tokyo, Japan). An emulsifiable concentrate-type formulation of MeSA (MeSA 20 EC) was prepared by mixing 20% (w/w) MeSA, 20% (w/w) propylene glycol mono methyl ether (PM), 20% (w/w) CR-MOC25, and 40% (w/w) methylated soybean oil (MOS2). ASM (>99.4% purity) was supplied by Syngenta Korea (Seoul, Republic of Korea). A suspension concentrate-type formulation of ASM (ASM 5 SC) was prepared by mixing 5.03% (w/w) ASM, 2% (w/w) CR-KSP40M, 10% (w/w) propylene glycol, 0.1% (w/w) xanthan gum, 0.1% (w/w) defoamer, and 82.77% (w/w) water.

The PWD-causing nematode, *B. xylophilus*, was obtained from the National Institute of Forest Science (Seoul, South Korea). It was isolated from the infected tissues of red pine (*Pinus densiflora*) and is highly virulent to various species of the genus *Pinus*. For inoculum preparation, *B. xylophilus* was inoculated on fungal hyphae of *Botrytis cinerea* cultured on potato dextrose agar incubated at 25 °C for 1 week. The nematodes were then isolated from the culture medium by the Baermann funnel method [27]. A suspension of nematodes was adjusted to a concentration of 20,000 nematodes/mL in distilled water. *P. densiflora* seedlings were purchased from Daelim seedling farm, Okcheon, South Korea.

### 2.2. Pine Seedling Assay

MeSA and ASM formulations were dissolved in water at concentrations of 0.1 mM and 0.05 mM, respectively, then, 5 mL was foliar sprayed onto 3-year-old *P. densiflora* seedlings. The treatment was conducted twice with a 1-week interval. One week after treatment, pine seedlings were inoculated with PWN, as described below. A small slit was made using a surface-sterilized knife, and a small piece of absorbent cotton was inserted into the slit. A water suspension of nematodes (2000 nematodes/100 μL) was pipetted onto the absorbent cotton and then wrapped with Parafilm M (Heathrow Scientific, Vernon Hills, IL, USA) to prevent drying [28]. Distilled water containing 250 μg/mL Tween 20 was used as an untreated control and 5 replicates were maintained for each treatment. Disease severity in treated seedlings was evaluated at 30 days post-inoculation, according to a scale from 0 to 5, in which 0 = healthy seedlings with normal green needles, 1 = < 20% brown needles, 2 = 20–39% brown needles, 3 = 40–59% brown needles, 4 = 60–79% brown needles and bending of the seedlings terminal shoots, and 5 = 80–100% of brown needles and wilting of the whole seedling [22]. The seedling assay was conducted twice with 5 replicates for each treatment.

### 2.3. DNA Extraction, Metagenomic Sequencing, and 16S Library Preparation

Pine seedlings were detached from the pots, the roots were vigorously shaken to separate soils that are not tightly attached to the roots. A composite sample of approximately 10 g from roots with closely adhering soil particles were collected from different sides and depths of each seedling and mixed well. The collected sample from each seedling representing 1 replicate was dissolved in 20 mL of sterile distilled water, vigorously vortexed and centrifuged for 15 min at 10,000 *g* at 4 °C to remove excessive water. From the pellets, 250 mg were used for bacterial microbiome DNA extraction using the PowerSoil^®^ DNA Isolation Kit (MO BIO Laboratories, Carlsbad, CA, USA), following the manufacturer’s instructions. The obtained bacterial microbiome DNA was checked for concentration and quality using a NanoDrop2000 spectrophotometer (Thermo Fisher Scientific, Wilmington, USA) and by agarose gel electrophoresis. Qualified samples were then stored in Tris-EDTA buffer solution at −20 °C until use.

The metagenomic was based on high-throughput sequencing of the V3 and V4 variable regions of the 16S rRNA (amplicon size 350–500 bp) using the Herculase II fusion DNA polymerase Nextera XT Index Kit V2 in an Illumina^®^ MiSeq^®^ platform at Macrogen (Seoul, South Korea) using the primer pair:

(F), 5′-TCGTCGGCAGCGTCAGATGTGTATAAGAGACAGCCTACGGGNGGCWGCAG-3′;

(R), 5′-GTCTCGTGGGCTCGGAGATGTGTATAAGAGACAGGACTACHVGGGTATCTAATCC-3′.

The fast length adjustment of short reads (FLASH; http://ccb.jhu.edu/software/FLASH/) was used for merging the obtained paired-end reads [29]. Following purification and trimming of adaptors and low quality or short reads, clustering and annotation were performed using the CD-HIT-OTU-MiSeq and UCLUST algorithm, and qualified sequences were arranged into the respective operational taxonomic units (OTUs) at a cut off value of 97% using the Greengenes database [30,31,32]. The quantitative insights into microbial ecology version 2 (QIIME2) pipeline was used for the microbiome analysis including diversity statistics and taxonomic assignments of the obtained OTUs [33]. The obtained sequences were deposited as a sequence read archive in a database of the National Center for Biotechnology Information under the BioProject ID PRJNA607851.

### 2.4. Statistical Analysis

Seedling assays were conducted with 5 replicates per treatment, and data of disease severity were analyzed by the Statistical Analysis Systems (SAS Institute, Cary, NC, USA), using analysis of variance performed by GLM procedures, and the means were separated using the least significant difference (LSD) test at *p* < 0.05. Bacterial microbiome diversity statistics were performed using the QIIME2 scripts, R software (version 3.1.3), and the PAleontological STatistics software package (PAST) version 3.23 [34]. The principle coordinate analysis (PCoA) was performed based on the Bray-Curtis, unweighted and weighted UniFrac distances.

## 3. Results

### 3.1. Effect of Chemical Inducers on PWD Severity

**Figure 1:**
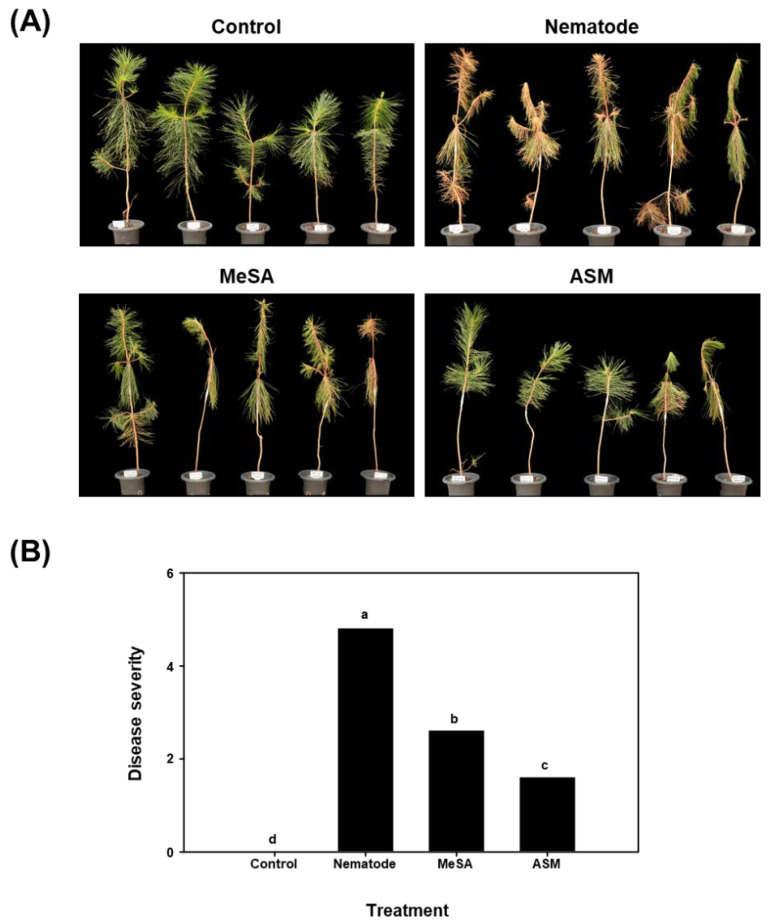
Effect of resistance-inducing chemical elicitors, methyl salicylic acid (MeSA), and acibenzolar-s-methyl (ASM), on the seedlings with pine wilt disease (PWD). (**A**) Photographs showing the PWD symptoms developed by pine (*Pinus densiflora*) seedlings treated with resistance-inducing chemical elicitors. Untreated seedlings served as the negative control whereas, nematode inoculated seedlings served as the positive control. (**B**) Bar graphs represent the mean PWD severity from five seedlings per treatment; different lowercase letters on the bars indicate a significant difference according to the least significant difference test at *p* < 0.05. Evaluation of disease severity was conducted 30 days post-inoculation with the pine wood nematode *Bursaphelenchus xylophilus*.

As shown in the photographs in Figure 1, PWD symptoms including wilting and browning of needles were clearly observed in the nematode-inoculated seedlings compared to that in the uninoculated controls. In the seedlings treated with resistance-inducing chemical elicitors, the severity of PWD symptoms was reduced compared to those in the nematode-inoculated controls. ASM-treated seedlings showed less severe symptoms compared to MeSA-treated seedlings (Figure 1A). The estimate of disease severity confirmed the observed differences between treatments. Specifically, PWD severity was significantly reduced (*p* < 0.05) in the seedlings treated with MeSA or ASM compared to that in the untreated controls. Moreover, treatment with ASM resulted in further significant reduction in disease severity compared to that after MeSA treatment (Figure 1B).

### 3.2. Sequence Analysis and Diversity in the Rhizosphere Bacterial Community in Different Pine Samples

A total of 2,192,402 reads with an average of 137,025 reads per sample were obtained by a high-throughput sequencing of 16 pine seedling rhizosphere samples. A summary of the total base count, reads, GC%, Q20%, and Q30% for each sample is shown in Table S1. After screening of low-quality, chimeras, or short reads using CD-HIT-OTU, 287,911 total clean reads were obtained with an average of 17,994 ± 2,493 reads per sample, ranging from a minimum of 14,661 to a maximum of 21,471 reads.

Rarefaction analysis from the obtained OTUs versus the number of sequence reads indicated satisfactory sequencing depth for all the samples as a near-plateau was reached at around 9000 reads, concluding that the major part of sample diversity had been detected (Figure S1).

There were no significant differences in richness or diversity of the bacterial communities within the different groups of pine seedling samples, as based on the OTUs number, Chao1, Shannon, and inverse Simpson indices (Table 1).

**Figure 2:**
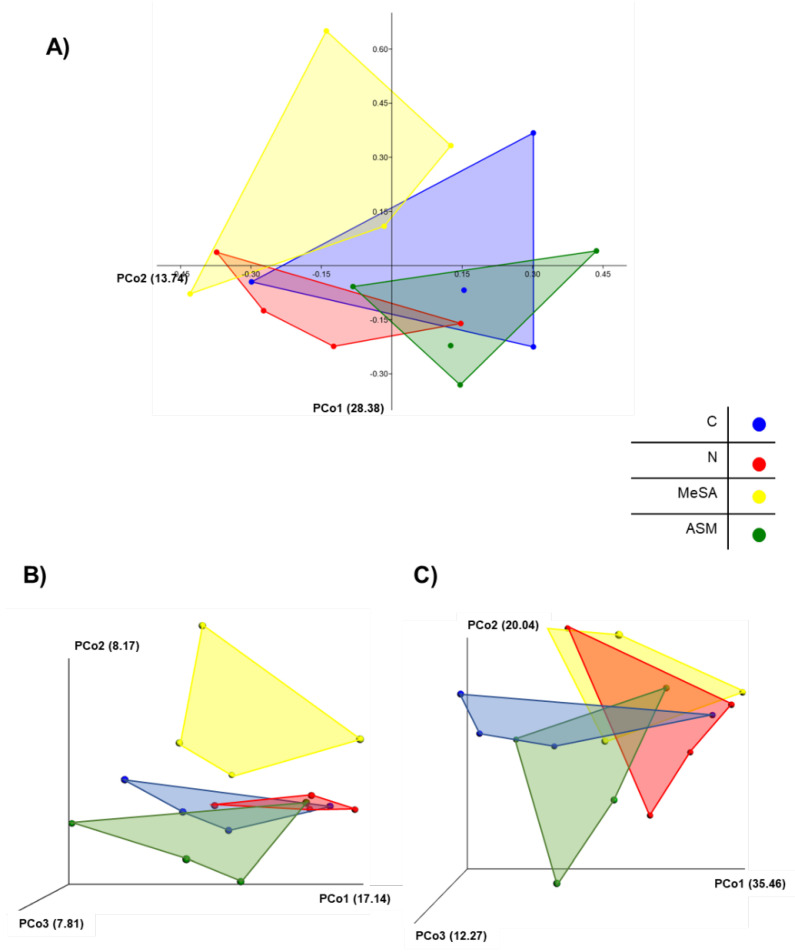
Principal coordinate analysis (PCoA) of the (**A**) Bray-Curtis, (**B**) unweighted UniFrac, and (**C**) weighted UniFrac distances from pine rhizosphere microbiome samples. C, negative control; N, nematode-inoculated; MeSA, nematode-inoculated after treatment with methyl salicylic acid; ASM, nematode-inoculated after treatment with acibenzolar-s-methyl.

With regard to the beta diversity between the different groups of pine rhizosphere, PCoA revealed slight distinct clustering, particularly in the pine seedling rhizosphere treated with MeSA compared to the negative control, the nematode-inoculated control and the pines treated with ASM according to Bray-Curtis and unweighted UniF-rac distances, which indicated a slight shift in the microbial structure in the MeSA-treated samples compared to the other groups (Figure 2A,B). When the weighted UniFrac distance was used to evaluate the abundance of the detected taxa, similarities were observed between the MeSA-treated pine samples and the nematode-treated control, whereas the ASM-treated samples were further separated from the other samples (Figure 2C).

### 3.3. Comparative Analysis of the Structure of Rhizosphere Microbial Communities in Different Pine Samples

**Figure 3:**
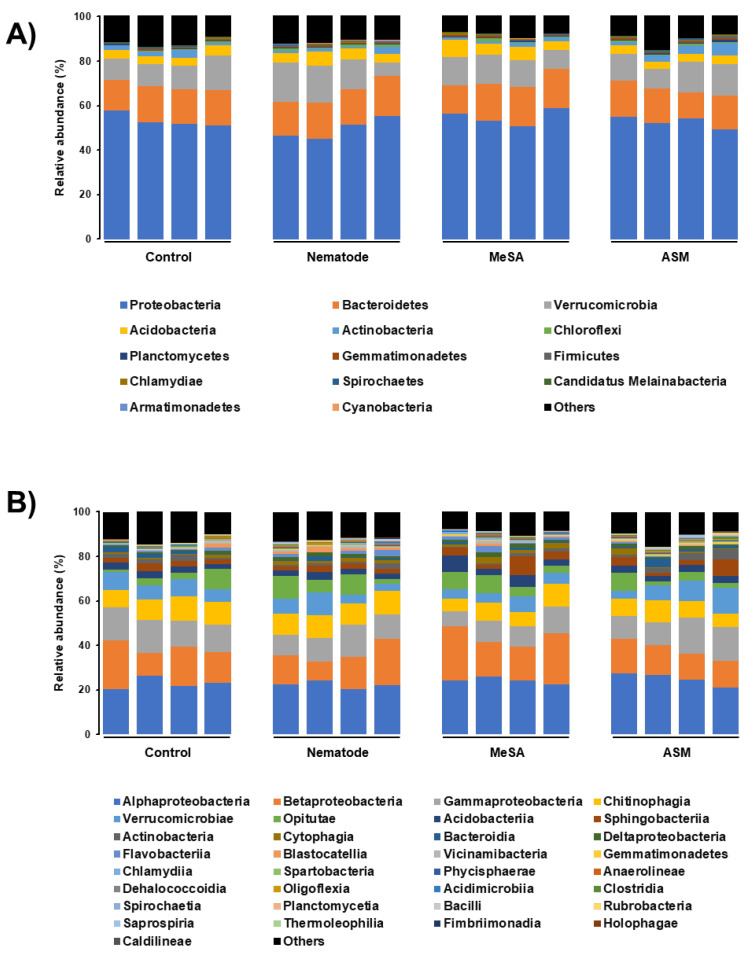
Stacked bar graphs representing the structure of pine rhizosphere microbiota at the phylum (**A**) and class (**B**) levels. Samples were treated with methyl salicylic acid (MeSA) or acibenzolar-s-methyl (ASM), followed by inoculation with the nematode, and compared to nematode-treated and control samples.

The stacked bar graphs in Figure 3, representing the relative abundance of the detected microbial taxa, at the phylum and class levels, revealed a similar community structure with no apparent difference in the dominant microbial groups (Figure 3). However, a deeper comparative look into the community structure of the microbial taxa with relatively low abundances revealed that the Firmicutes phylum and the Gammaproteobacteria, Chitinophagia, and Oligoflexia classes had a significantly lower (*p* < 0.05) abundance on the pine samples treated with MeSA than that on the control pine groups (Figure S2). The relative abundance of Planctomycetes and Spirochaetes phyla on the pine samples treated with ASM, was significantly higher (*p* < 0.05) than that on the corresponding controls, whereas Cyanobacteria and Chitinophagia were significantly lower (*p* < 0.05) than that on the control pine samples (Figure S3). The microbial taxa with significantly different relative abundance at the phylum, class, order, and family levels in the pine samples treated with MeSA or ASM, as compared to the other pine groups, are shown in Figures S2 and S3.

**Figure 4:**
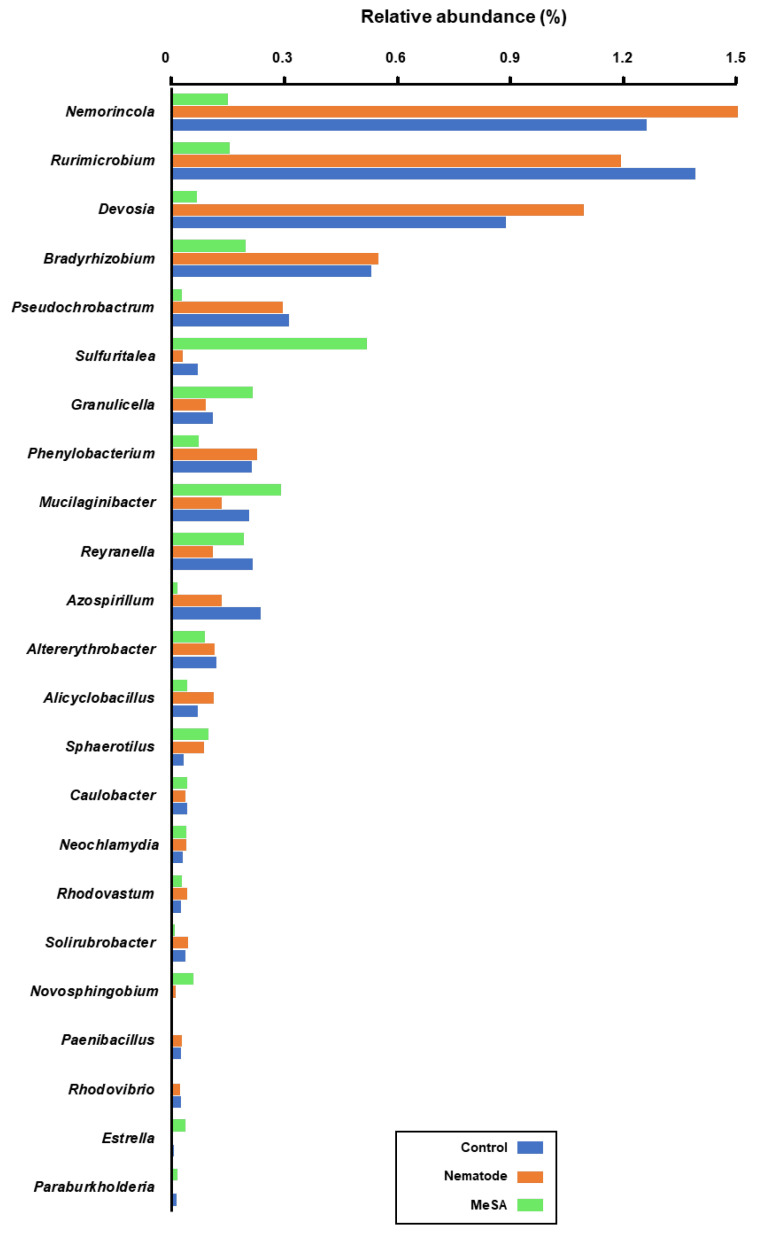
The relative abundance of bacterial genera in the rhizosphere of pine seedlings treated with methyl salicylic acid (MeSA) and followed by inoculation with the nematode, as compared to nematode-inoculated or control seedlings. Bacterial genera with significantly different (*p* < 0.05) relative abundance after treatment with MeSA compared to controls are shown in the graph. The bars represent the mean values (*n* = 4).

At the genus level, *Nemorincola*, *Rurimicrobium*, *Devosia*, *Bradyrhizobium*, *Pseudochrobactrum*, *Azospirillum*, *Altererythrobacter*, *Phenylobacterium*, *Alicyclobacillus*, *Reyranella*, *Rhodovastum*, *Solirubrobacter*, *Paenibacillus*, and *Rhodovibrio* showed significantly lower (*p* < 0.05) relative abundance in the pine samples treated with MeSA, whereas *Sulfuritalea*, *Granulicella*, *Mucilaginibacter*, *Sphaerotilus*, *Caulobacter*, *Neochlamydia*, *Reyranella terrae*, *Novosphingobium*, *Estrella*, and *Paraburkholderia* showed significantly high relative abundance in the MeSA-treated pine samples compared to that in the control groups (Figure 4).

**Figure 5:**
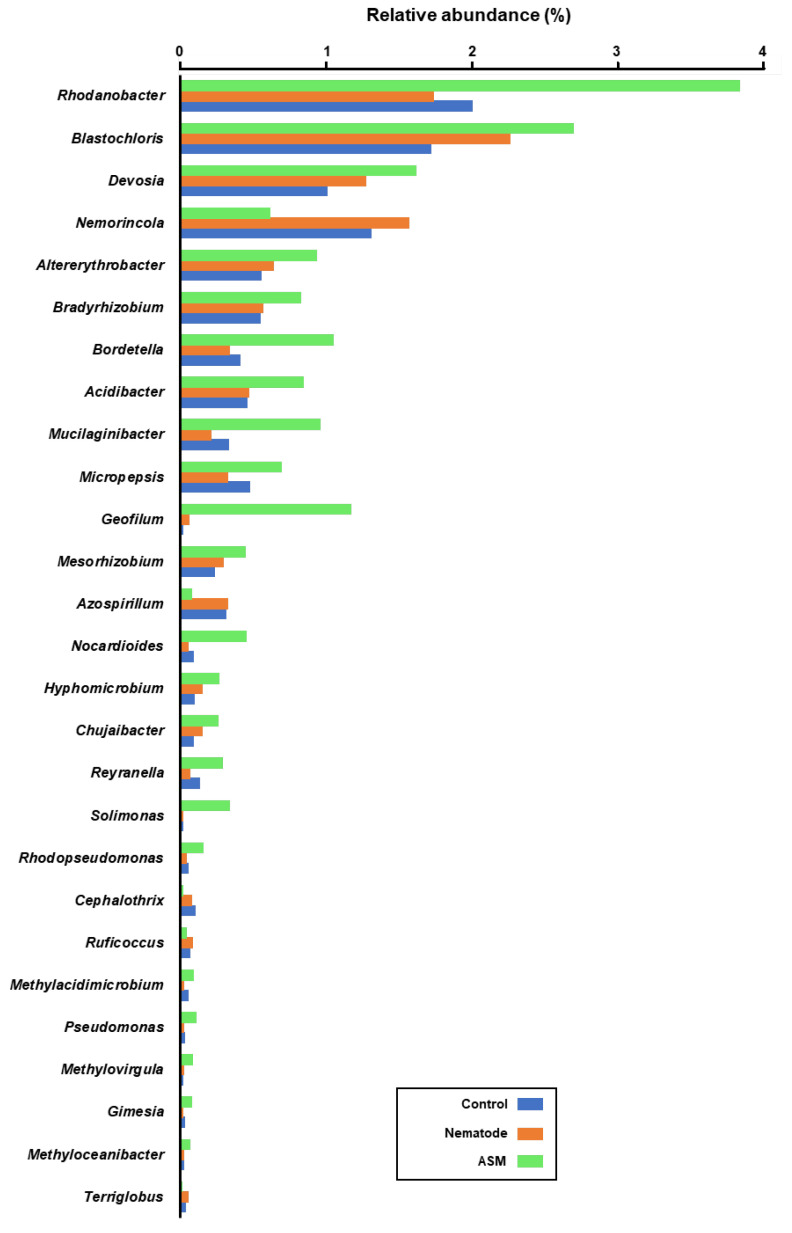
The relative abundance of bacterial genera in the rhizosphere of pine seedling treated with acibenzolar-s-methyl (ASM) and followed by inoculation with the nematode, as compared to nematode-inoculated or control seedlings. Only bacterial genera that existed in significantly different (*p* < 0.05) relative abundance after treatment with ASM compared to the control are shown in the graph. The bars represent the mean values (*n* = 4).

The bacterial genera with significantly higher relative abundance in the ASM-treated pine seedlings than in the control were as follows: *Rhodanobacter, Devosia, Altererythrobacter, Bradyrhizobium, Bordetella, Acidibacter, Mucilaginibacter, Micropepsis, Geofilum, Mesorhizobium, Nocardioides, Hyphomicrobium, Chujaibacter, Solimonas, Reyranella, Rhodopseudomonas, Methylacidimicrobium, Pseudomonas, Methylovirgula, Gimesia*, and *Methyloceanibacter*. On the contrary, *Nemorincola, Azospirillum, Cephalothrix, Ruficoccus*, and *Terriglobus* showed significantly lower relative abundance in the pine seedlings treated with ASM than in the controls (Figure 5).

**Figure 6:**
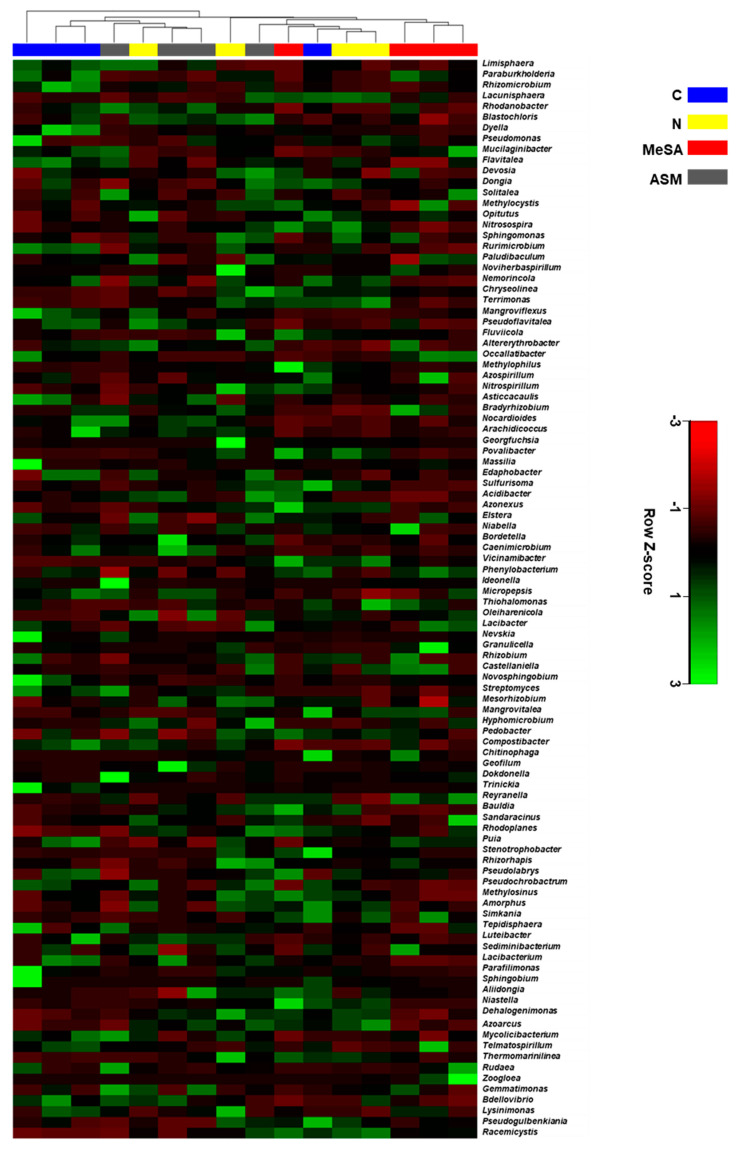
Heatmap of the average linkage hierarchical clustering, based on the Manhattan distance measurement of dominant 100 bacterial genera in the rhizosphere of pine seedlings. C, negative control; N, nematode-inoculated; MeSA, nematode-inoculated pine seedlings treated with methyl salicylic acid; ASM, nematode-inoculated pine seedlings treated with acibenzolar-s-methyl. The Z-Score indicates the relative abundance of bacterial genera in each row following heatmap standardization.

The heatmap shown in Figure 6 represents the average linkage hierarchical clustering based on the Manhattan distance of the dominant 100 bacterial genera in the four groups of pine seedlings. Although there were no clear distinctions between the different groups, three out of four pine samples treated with MeSA showed a distinctive cluster separated from the control and the ASM-treated pine groups (Figure 6).

## 4. Discussion

Conventionally, chemical nematicides have been effectively used for controlling plant parasitic nematode infections. However, due to increased understanding of their potential environmental effects, as well as human health-related concerns, restrictions are being made on their use, and several chemical nematicides have been withdrawn from the market due to their deleterious effects [35]. Hence, research on safer alternatives—such as biological control and induced resistance—is encouraged. In this study, treatment with chemical resistance-inducing elicitors resulted in reduced PWD severity, with ASM showing higher effectiveness than MeSA. Strategies based on the induction of resistance against plant-parasitic nematodes using chemical elicitors proved considerably successful and were demonstrated to be an efficient and eco-friendly way for controlling plant-parasitic nematodes [35,36]. In tomato plants, treatment with SA and ASM was shown to significantly reduce the reproduction and infestation of root-knot nematodes compared to that after treatment with MeSA [23]. Regarding PWN, chemical inducers such as γ-aminobutyric acid, were found to trigger the expression of certain pathogenesis-related genes and, consequently, PWD severity was reduced in treated seedlings. Furthermore, specific pine endophytic bacterial species were utilized for induction of resistance against PWD [22].

Research on the induction of resistance against plant-parasitic nematodes has always focused on the control mechanisms, efficacy, and physiological changes in host plants [24,35]. Nevertheless, the environmental conditions, mainly the biotic factors, surrounding hosts and pathogens are key players for the development of the disease. The microbial communities play a critical role in the pine tree ecosystem, and their characterization will advance the understanding of disease development and may pave the way for its possible management [16]. The rhizosphere microbial community is fundamental for healthy plant growth due to its remarkable ability to synthesize diverse groups of secondary metabolites in response to different abiotic and biotic stresses [37]. Notably, the structure of the rhizosphere microbial community relies on several factors. The exudates secreted by the plant roots exert a major influence on the microbial composition of adjacent soil [38]. It has been reported that approximately 5% to 21% of all photosynthetically fixed carbon is translocated to the rhizosphere through root exudates [38,39]. It could be suggested that treatment with resistance-inducing chemical elicitors may cause changes in the physiology of the host trees, leading to variations in the metabolites and root exudates, which in turn may result in shifts in the composition of rhizosphere microbial communities.

Therefore, in this study, resistance-inducing chemical elicitors (MeSA and ASM) were tested not only for their control efficacy against PWD, but also for their influence on the microbial composition of the rhizosphere and roots of pine seedlings. In this study, no significant differences were found in alpha diversity between the pine groups. However, beta diversity showed a partial separation between samples, particularly those treated with MeSA. The microbial profiles showed similarities in the dominant taxa between the different samples, while treatments with MeSA and ASM induced differences in bacterial taxa of relatively low abundance.

Among bacteria displaying a significant increase in relative abundance in pine seedlings treated with ASM, there were members of the *Rhodanobacter, Devosia, Bradyrhizobium, Acidibacter, Mesorhizobium,* and *Hyphomicrobium* genera. Members of the genus *Rhodobacter* and *Hyphomicrobium* are known to have several important ecological roles such as denifirication and herbicide degradation [40,41,42]. In particular, *Hyphomicrobium denitrificans* was shown to possess genes encoding the full set of enzymes required for the complete denitrification process and has been found to biodegrade dimethyl sulfoxide [42,43]. Furthermore, *Bradyrhizobium*, *Mesorhizobium,* and *Devosia* spp. were reported as efficient nitrogen fixers and, in some cases, as root modulators [44,45,46]. A strain of *Mesorhizobium amorphae*, in addition to being a nitrogen-fixing and growth-promoting rhizobacterium, has the ability to perform copper phytoremediation in contaminated soils [47]. *Acidibacter ferrireducens* is an acidic condition-tolerant species that is capable of reducing ferric iron [48].

On the contrary, treatment with MeSA resulted in an increase in the sulphur-oxidizing *Sulfuritalea* and acid-tolerant *Granulicella* [49,50]. However, unlike ASM, MeSA treatment significantly reduced the nitrogen-fixing and root modulators, *Devosia* and *Bradyrhizobium* spp., which suggested a more beneficial impact of ASM compared to that of MeSA on the rhizosphere microbiota to support healthy plant growth.

Intriguingly, chitinolytic bacteria belonging to the Chitinophagaceae family, including members of the *Nemorincola* and *Rurimicrobium* genera, were significantly reduced in response to treatment with both resistance-inducing chemical elicitors, ASM and MeSA. Moreover, MeSA treatment resulted in a significant reduction in the relative abundance of *Paenibacillus*, which encompasses members previously shown to exhibit chitinolytic activity [51]. It is possible that the increase in the plant chitinase production, coupled with the reduction in the nematode population, is the reason for the altered microenvironment, rendering it less favorable for such chitinolytic microbes. Consistently, it has been reported that chemical elicitors may increase the expression of endochitinase-related genes such as PR-3 family members, class I chitinase, and class IV chitinase. Since chitin is a main component of nematodes and their eggshells, the enzymes encoded by the latter genes could inhibit nematodes by inducing structural changes in their cuticle and inhibiting egg hatching [22,52,53]. It was also reported that supplementing soils with chitin associates with an increase in bacterial populations with chitinolytic activity [54].

The present findings should be evaluated in light of specific limitations that could be addressed in future studies. The analysis of microbial composition could have been more informative if the microbial composition of the endorhiza, representing the root internal tissues, was separately examined from that of the rhizosphere. In addition, the possible influence of the resistance-inducing chemical elicitors on pine endophytic microbial communities should be addressed, as these communities were reported to play a critical role in PWD development [16,22].

Taken together, the results of this study indicated that treatment with the resistance-inducing chemical elicitors, MeSA and ASM, resulted in significant reduction in PWD severity, with ASM being more effective than MeSA at suppressing PWD symptoms. In addition, no significant differences were observed in the abundance of the dominant microbial populations, whereas significant changes were detected in the microbial communities with low relative abundance. The latter finding should be taken into account in future practical applications of the chemical elicitors. Treatment with ASM resulted in a significant increase in the proportion of several rhizosphere microbial taxa that were previously reported to play essential ecological and plant growth-promoting roles. The data presented in the current study provide important and novel information about possible alterations in the rhizosphere microbiota in response to foliar treatment with resistance-inducing chemical elicitors.

## Figures and Tables

**Table 1:** Alpha diversity indices in rhizosphere microbiome of pine seedlings.

Treatment	OTUs	Chao1	Shannon	Inverse Simpson	Good’s Coverage
C	1120.75 ± 151.33	1328.54 ± 157.73	7.76 ± 0.49	0.98 ± 0.01	0.99 ± 0.00
N	1265.75 ± 143.47	1464.90 ± 141.34	8.28 ± 0.20	0.99 ± 0.00	0.99 ± 0.00
MeSA	1119.25 ± 123.72	1313.64 ± 137.90	8.06 ± 0.32	0.99 ± 0.00	0.99 ± 0.00
ASM	1078 ± 206.98	1245.55 ± 224.81	7.99 ± 0.33	0.99 ± 0.00	0.99 ± 0.00

Values are the means ± standard deviation (*n* = 4). OTUs, operational taxonomic units; C, control pine seedlings; N, nematode-inoculated pine seedlings; MeSA, nematode-inoculated pine seedlings treated with methyl salicylic acid; ASM, nematode-inoculated pine seedlings treated with acibenzolar-s-methyl.

## Supplementary Materials

The following materials are available online at https://www.mdpi.com/2076-2607/8/6/884/s1: Table S1: results of high-throughput sequencing representing the total bases, read count, GC%, Q20%, and Q30%; Figure S1: rarefaction curves of the obtained 16S rRNA sequence reads against the assigned operational taxonomic units (OTUs) for evaluation of the sequencing depth from pine rhizosphere samples. C, negative control; N, nematode-inoculated; MeSA, nematode-inoculated pine seedlings and treated with methyl salicylic acid; ASM, nematode-inoculated pine seedlings treated with acibenzolar-s-methyl; Figure S2: bar graphs representing the relative abundance of bacterial taxa at (A) phylum, (B) class, (C) order, and (D) family levels, present at significantly (*p* < 0.05) different levels in pine seedlings treated with methyl salicylic acid (MeSA), followed by inoculation with the nematode, compared to nematode-inoculated or control seedlings; Figure S3: bar graphs representing the relative abundance of bacterial taxa at (A) phylum, (B) class, (C) order, and (D) family levels, present at significantly (*p* < 0.05) different levels in pine seedlings treated with acibenzolar-s-methyl (ASM), followed by inoculation with the nematode, compared to nematode-treated or control seedlings.
