# Supplementary material for: Influence of Resistance-Inducing Chemical Elicitors against Pine Wilt Disease on the Rhizosphere Microbiome
Source: Microorganisms. 2020 Jun 11;8(6):884. doi: 10.3390/microorganisms8060884 (PMC7356868; doi:10.3390/microorganisms8060884)
Supplement: Supplementary file 1 [file microorganisms-08-00884-s001.zip › Suppl/microorganisms-825024 suppl figures resub.pptx]

## Slide 1
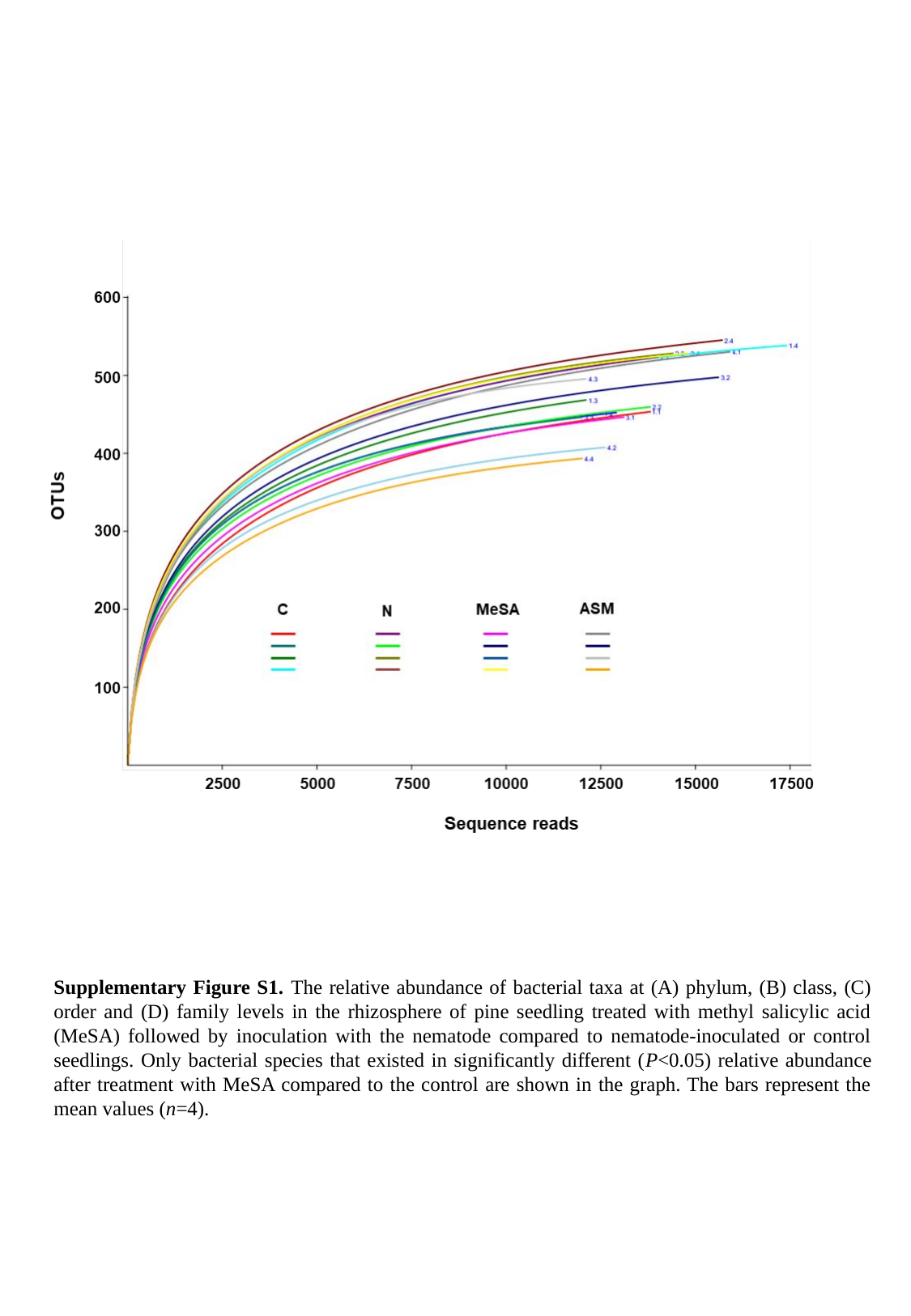

Supplementary Figure S1. The relative abundance of bacterial taxa at (A) phylum, (B) class, (C) order and (D) family levels in the rhizosphere of pine seedling treated with methyl salicylic acid (MeSA) followed by inoculation with the nematode compared to nematode-inoculated or control seedlings. Only bacterial species that existed in significantly different (P<0.05) relative abundance after treatment with MeSA compared to the control are shown in the graph. The bars represent the mean values (n=4).

## Slide 2
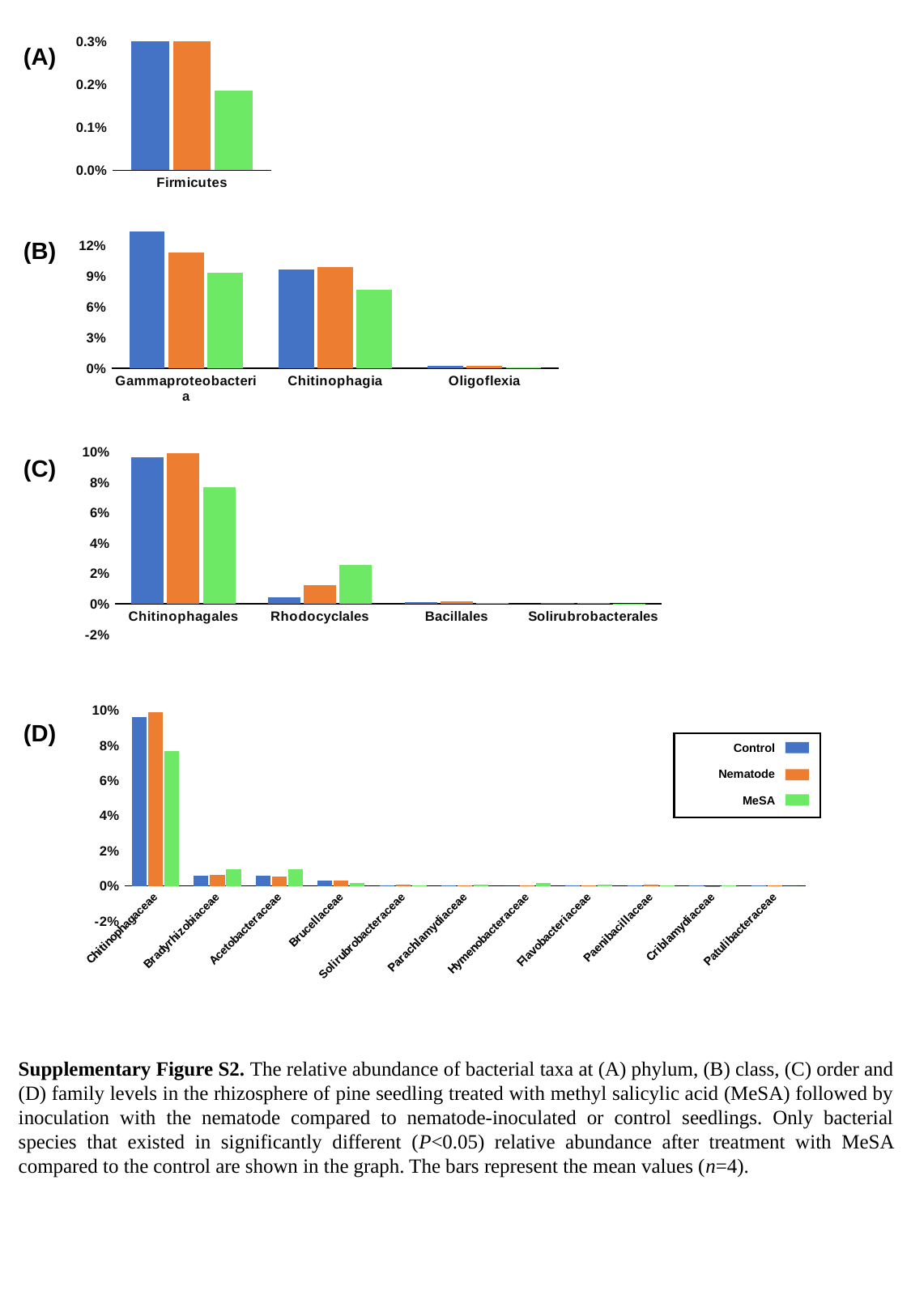

### Chart
| Category | C | N | NM |
|---|---|---|---|
| Firmicutes | 0.0033889690228800003 | 0.0034195527281249997 | 0.0018450490822825 |(A)
### Chart
| Category | C | N | NM |
|---|---|---|---|
| Gammaproteobacteria | 0.133885449614 | 0.11297823167812499 | 0.0934363967281 |
| Chitinophagia | 0.0961830931108 | 0.0990319194891 | 0.07658134249137499 |
| Oligoflexia | 0.0023572196538675 | 0.0024637910712125 | 0.0010802983623975 |(B)
### Chart
| Category | C | N | NM |
|---|---|---|---|
| Chitinophagales | 0.0961830931108 | 0.0990319194891 | 0.07658134249137499 |
| Rhodocyclales | 0.0044023624536765 | 0.012563245704575 | 0.025743433948032503 |
| Bacillales | 0.0013762813378332501 | 0.00193909968580125 | 0.0007000875590200001 |
| Solirubrobacterales | 0.000755296433536 | 0.000702667295971 | 0.000254148174295675 |(C)
### Chart
| Category | C | N | NM |
|---|---|---|---|
| Chitinophagaceae | 0.0961830931108 | 0.0990319194891 | 0.07658134249137499 |
| Bradyrhizobiaceae | 0.0059059383028975 | 0.0062303337158725 | 0.0093875827991 |
| Acetobacteraceae | 0.0059535459221725 | 0.0053462615635825 | 0.0095613727559475 |
| Brucellaceae | 0.0031448525534074996 | 0.0029841652471275003 | 0.0015406612357575001 |
| Solirubrobacteraceae | 0.000364588156436625 | 0.0005610580630542501 | 0.00013569499795175 |
| Parachlamydiaceae | 0.000352003102148325 | 0.00043739351643324996 | 0.000909287182669 |
| Hymenobacteraceae | 0.0 | 0.00024220026671827502 | 0.0016292198152439998 |
| Flavobacteriaceae | 0.00016785538855025 | 0.00034625174648175 | 0.00094322941433975 |
| Paenibacillaceae | 0.00044726324149225007 | 0.00053987417538775 | 8.887483744675e-05 |
| Criblamydiaceae | 6.167522639815e-05 | 4.577916132575e-05 | 0.00041774893049000006 |
| Patulibacteraceae | 0.00017073131383132502 | 9.141867553275e-05 | 0.0 |(D)
Control
Nematode
MeSA
Supplementary Figure S2. The relative abundance of bacterial taxa at (A) phylum, (B) class, (C) order and (D) family levels in the rhizosphere of pine seedling treated with methyl salicylic acid (MeSA) followed by inoculation with the nematode compared to nematode-inoculated or control seedlings. Only bacterial species that existed in significantly different (P<0.05) relative abundance after treatment with MeSA compared to the control are shown in the graph. The bars represent the mean values (n=4).

## Slide 3
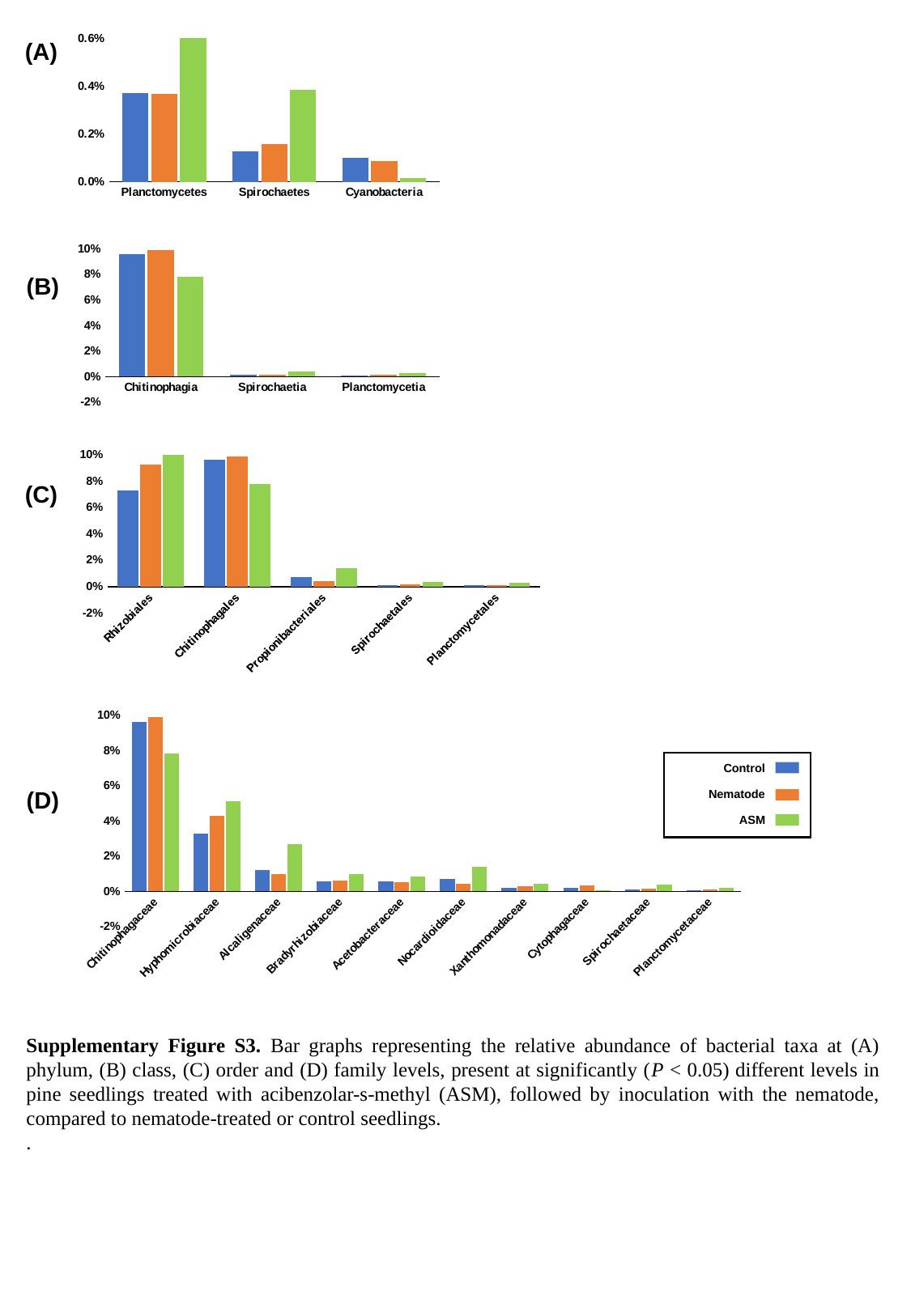

### Chart
| Category | MAN.4 | NMT.MAC.4 | NMT.MAA.4 |
|---|---|---|---|
| Planctomycetes | 0.0037001345521374997 | 0.00367456028929 | 0.0061471792006125 |
| Spirochaetes | 0.001267138053523 | 0.00158987990892125 | 0.0038362266433425 |
| Cyanobacteria | 0.0009956295504287502 | 0.0008579131359625 | 0.00016871771936212502 |(A)
### Chart
| Category | MAN | NMT.MAC | NMT.MAA |
|---|---|---|---|
| Chitinophagia | 0.0961830931108 | 0.0990319194891 | 0.07819139326454999 |
| Spirochaetia | 0.001267138053523 | 0.00158987990892125 | 0.0038362266433425 |
| Planctomycetia | 0.00117673605201825 | 0.0012229769570542498 | 0.0029549184950199998 |(B)
### Chart
| Category | MAN | NMT.MAC | NMT.MAA |
|---|---|---|---|
| Rhizobiales | 0.0729414637021 | 0.09251730436437501 | 0.104009511452475 |
| Chitinophagales | 0.0961830931108 | 0.0990319194891 | 0.07819139326454999 |
| Propionibacteriales | 0.0070436063980525 | 0.00411715178856 | 0.0140557038914675 |
| Spirochaetales | 0.001267138053523 | 0.0015593604680375001 | 0.0038116300278375004 |
| Planctomycetales | 0.00117673605201825 | 0.0012229769570542498 | 0.0029549184950199998 |(C)
### Chart
| Category | MAN | NMT.MAC | NMT.MAA |
|---|---|---|---|
| Chitinophagaceae | 0.0961830931108 | 0.0990319194891 | 0.07819139326454999 |
| Hyphomicrobiaceae | 0.032664031560325 | 0.042999992560175004 | 0.05122643961855 |
| Alcaligenaceae | 0.012329278419865 | 0.009962733162335001 | 0.02667428284685 |
| Bradyrhizobiaceae | 0.0059059383028975 | 0.0062303337158725 | 0.009764964277655 |
| Acetobacteraceae | 0.0059535459221725 | 0.0053462615635825 | 0.0084154436022975 |
| Nocardioidaceae | 0.0070436063980525 | 0.00411715178856 | 0.0140557038914675 |
| Xanthomonadaceae | 0.0022631058486225 | 0.0028894295127575 | 0.0045372338859124995 |
| Cytophagaceae | 0.0018091645972714999 | 0.003476461896725 | 0.00088428878064575 |
| Spirochaetaceae | 0.001267138053523 | 0.0015593604680375001 | 0.0038116300278375004 |
| Planctomycetaceae | 0.00071207039750075 | 0.0009073206276845 | 0.002028794670885 |
Control
Nematode
ASM
(D)
Supplementary Figure S3. Bar graphs representing the relative abundance of bacterial taxa at (A) phylum, (B) class, (C) order and (D) family levels, present at significantly (P < 0.05) different levels in pine seedlings treated with acibenzolar-s-methyl (ASM), followed by inoculation with the nematode, compared to nematode-treated or control seedlings.
.
