# Supplementary material for: Influence of Resistance-Inducing Chemical Elicitors against Pine Wilt Disease on the Rhizosphere Microbiome
Source: Microorganisms. 2020 Jun 11;8(6):884. doi: 10.3390/microorganisms8060884 (PMC7356868; doi:10.3390/microorganisms8060884)
Supplement: Supplementary file 1 [file microorganisms-08-00884-s001.zip › Suppl/microorganisms-825024 SuppTable.doc]

**MeSA**

**Table S1.** Results of the high throughput metagenomics sequencing representing the total bases, read count, GC%, Q20% and Q30%

| Sample | Total bases | Read count | GC (%) | Q20 (%) | Q30 (%) |
| --- | --- | --- | --- | --- | --- |
| C1 | 64,551,305 | 142,674 | 54.04 | 98.37 | 94.57 |
| C2 | 61,289,773 | 135,905 | 54.24 | 98.25 | 94.16 |
| C3 | 64,681,857 | 143,109 | 54.29 | 98.3 | 94.38 |
| C4 | 67,235,944 | 148,336 | 54.24 | 98.28 | 94.39 |
| N1 | 64,467,396 | 142,452 | 54.2 | 98.22 | 94.18 |
| N2 | 59,054,719 | 130,742 | 54.34 | 98.12 | 93.9 |
| N3 | 62,658,175 | 137,981 | 54.27 | 98.28 | 94.35 |
| N4 | 69,184,856 | 153,048 | 54.23 | 98.29 | 94.33 |
| MeSA1 | 55,748,266 | 123,086 | 54.38 | 98.35 | 94.5 |
| MeSA2 | 69,514,296 | 153,590 | 54.47 | 98.27 | 94.31 |
| MeSA3 | 53,056,354 | 117,337 | 54.19 | 98.34 | 94.44 |
| MeSA4 | 64,676,758 | 142,851 | 54.11 | 98.26 | 94.33 |
| ASM1 | 69,539,757 | 153,880 | 54.47 | 98.35 | 94.53 |
| ASM2 | 53,034,919 | 117,715 | 54.36 | 98.1 | 93.9 |
| ASM3 | 58,676,028 | 129,826 | 54.46 | 98.12 | 93.89 |
| ASM4 | 54,229,386 | 119,870 | 54.66 | 98.24 | 94.23 |

C, negative control; N, nematode inoculated; MeSA, MeSA treatment added nematode; ASM, ASM treatment added nematode;
